# A Micellar Formulation of Quercetin Prevents Cisplatin Nephrotoxicity

**DOI:** 10.3390/ijms22020729

**Published:** 2021-01-13

**Authors:** Alfredo G. Casanova, Marta Prieto, Clara I. Colino, Carmen Gutiérrez-Millán, Barbara Ruszkowska-Ciastek, Esther de Paz, Ángel Martín, Ana I. Morales, Francisco J. López-Hernández

**Affiliations:** 1 Institute of Biomedical Research of Salamanca (IBSAL), 37007 Salamanca, Spain; alfredogcp@usal.es (A.G.C.); martapv@usal.es (M.P.); ganda@usal.es (C.I.C.); carmengutierrez@usal.es (C.G.-M.); 2 Department of Physiology and Pharmacology, University of Salamanca, 37007 Salamanca, Spain; 3 Toxicology Unit, University of Salamanca, 37007 Salamanca, Spain; 4 Area of Pharmacy and Pharmaceutical Technology, Department of Pharmaceutical Sciences, University of Salamanca, 37007 Salamanca, Spain; 5 Department of Pathophysiology, Collegium Medicum in Bydgoszcz, Nicolaus Copernicus University in Torun, 85-796 Bydgoszcz, Poland; ruszkowska.basia@gmail.com; 6 High Pressure Processes Group, BioEcoUVa, Bioeconomy Research Institute, Department of Chemical Engineering and Environmental Technology, University of Valladolid, 47011 Valladolid, Spain; edepaz@gmail.com (E.d.P.); mamaan@iq.uva.es (Á.M.)

**Keywords:** cisplatin, nephrotoxicity, flavonoid, quercetin, nephroprotection, bioavailability, kidney, micelles, solubility, formulation

## Abstract

The antioxidant flavonoid quercetin has been shown to prevent nephrotoxicity in animal models and in a clinical study and is thus a very promising prophylactic candidate under development. Quercetin solubility is very low, which handicaps clinical application. The aim of this work was to study, in rats, the bioavailability and nephroprotective efficacy of a micellar formulation of Pluronic F127-encapsulated quercetin (P-quercetin), with improved hydrosolubility. Intraperitoneal administration of P-quercetin leads to an increased plasma concentration and bioavailability of quercetin compared to the equimolar administration of natural quercetin. Moreover, P-quercetin retains overall nephroprotective properties, and even slightly improves some renal function parameters, when compared to natural quercetin. Specifically, P-quercetin reduced the increment in plasma creatinine (from 3.4 ± 0.5 to 1.2 ± 0.3 mg/dL) and urea (from 490.9 ± 43.8 to 184.1 ± 50.1 mg/dL) and the decrease in creatinine clearance (from 0.08 ± 0.02 to 0.58 ± 0.19 mL/min) induced by the nephrotoxic chemotherapeutic drug cisplatin, and it ameliorated histological evidence of tubular damage. This new formulation with enhanced kinetic and biopharmaceutical properties will allow for further exploration of quercetin as a candidate nephroprotector at lower dosages and by administration routes oriented towards its clinical use.

## 1. Introduction

Drug nephrotoxicity is a serious medical and economic concern [1,2], with 25% of the 100 most-used drugs in intensive care units being nephrotoxic [3], and nephrotoxicity is also an important cause of candidate drop during the drug discovery process [4]. Cisplatin is a platinum-based antitumor agent frequently used in the treatment of a diversity of solid malignant neoplasms [5,6]. Nearly 30% of patients show evidence of nephrotoxicity during the first ten days following cisplatin administration, which poses an important limitation to its dosage and therapeutic effectiveness [5,6,7,8]. Acute cisplatin nephrotoxicity causes a tubulopathy derived from a 5-fold accumulation in the epithelial cells of the proximal tubule, with respect to its plasma concentration [9,10,11], and to a lesser extent in the distal tubule [12,13].

Cisplatin tubulopathy features electrolytic disturbances (mainly hypomagnesemia and hypokalemia) and acute kidney injury (AKI). AKI is a common syndrome characterized by an abrupt decline in glomerular filtration rate (GFR), severe azotemia, and, often, oliguria or anuria [7,14]. Additionally, endothelial dysfunction that increases renal vascular resistance and impairs autoregulation also contributes to cisplatin-induced AKI [9]. Ordinarily, AKI is a reversible condition, which nonetheless has a relevant impact on patient outcomes, including elevated in-hospital mortality (over 50% of cases among the critically ill), prolonged hospitalization, additional health care costs, and, in the middle- and long-term scenarios, increased risk of developing chronic kidney disease and of general and cardiovascular morbimortality [7,14]. At the subcellular and molecular levels, cisplatin tubular cytotoxicity is driven by mitochondrial injury, which curtails respiration, produces oxidative stress, and induces apoptotic and necrotic cell death and a deleterious inflammatory response [13,15,16]. Oxidative stress is recognized as a central mechanism of cisplatin cytotoxicity and nephrotoxicity [12,13,15,16,17], arising from an increased production of reactive oxygen species and a weakened endogenous antioxidant enzyme barrier [5,7,18].

Effective prophylactic measures for cisplatin nephrotoxicity pose an unmet clinical need towards improving its pharmaco-toxicological profile and maximizing its utility. Existing preventive strategies, including intensive hydration, have only demonstrated limited protection [6,19]. New strategies based on the co-administration of nephroprotectors are under development. Candidate nephroprotectors include magnesium formulations [6,9] and, most prominently, a variety of antioxidants [6,20,21]. Flavonoids are a family of nephroprotective polyphenolic products derived from vegetables, fruits, nuts, and wine, with strong antioxidant properties [22]. Quercetin is a featured flavonoid that exerts many beneficial effects [23,24], including scavenging of reactive oxygen species, suppression of platelet activation, endothelial protection, modulation of inflammation, inhibition of apoptosis, tumor suppression, and nephroprotection. Co-administration of quercetin alongside cisplatin therapy in an animal tumor model affords nephroprotection without interfering with the antitumor effect [25,26]. In fact, an updated meta-analysis identified quercetin as a very promising candidate as a nephroprotector for further clinical development [21]. Consistently, a recent clinical study reported a protective effect of quercetin on contrast-induced nephropathy (CIN) [27].

A strong limitation to the potential application of quercetin (shared by flavonoids in general) is its low hydrosolubility arising from its chemical structure and moieties, which reduce its absorption in the small gut, and thus its bioavailability and efficacy [23,24,28]. Interestingly, lipophobic glucose–quercetin conjugates (glucosides) are substantially more bioavailable than are the lipophilic aglycones, because the latter are less soluble in the intestinal lumen [24]. In fact, quercetin glucosides from onions show the highest absorption rate, and dietary fat enhances quercetin aglycone absorption in the small gut [29]. The very low hydrosolubility of quercetin not only impedes its clinical use by practical administration routes (i.e., oral, intravascular), but also curtails preclinical research. Nevertheless, in animal models, alternative routes (i.e., intraperitoneal) with drug suspensions can be used for proof-of-concept purposes [25,26].

Quercetin formulations with new carrier materials bearing improved hydrosolubility have been developed, whose biomedical properties need to be tested. Pluronic poloxamers are a class of carrier materials that host and enhance absorption of water insoluble compounds due to their ability to form micelles in aqueous environments [30]. Herein, we hypothesized that a micellar formulation of quercetin encapsulated with Pluronic F127, previously described [31], would retain the nephroprotective properties of natural quercetin, while offering improved biopharmaceutical characteristics for handling, formulation, and administration.

## 2. Results

A bioavailability and a nephroprotection study were carried out with the new micellar formulation of quercetin encapsulated with Pluronic F127 [31], and our traditional formulation of natural quercetin [25,26] (see below Materials and Methods). Whereas this latter was a suspension containing a tensoactive, the former was a saline solution with no extra additives. Our traditional formulation of natural quercetin was only apt for experimental purposes, precipitated when left still, and was more difficult to handle and inject. In contrast, the micellar formulation behaved as a solution and showed no usage inconveniences.

### 2.1. Bioavailability Study

**Figure 1:**
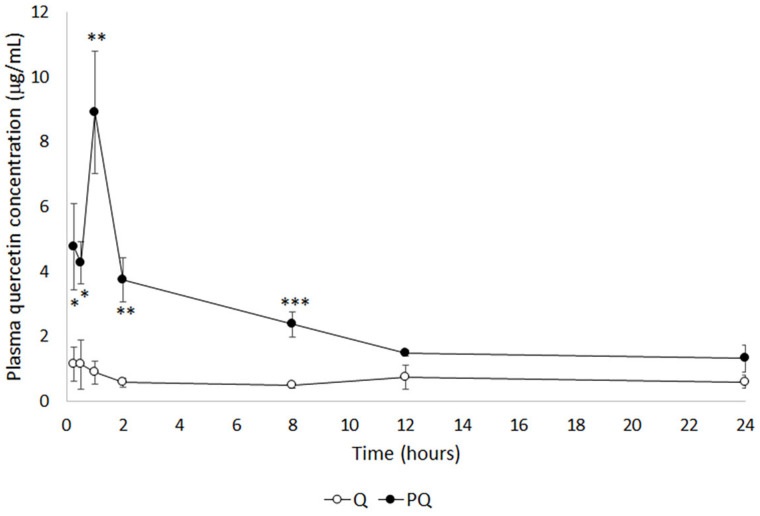
Evolution of the plasma concentration of quercetin after intraperitoneal administration of a single bolus of equimolar P-quercetin and natural quercetin. Values are expressed as the mean ± standard error of the mean (SEM) (*n* = 5 per group). * *p* < 0.05; ** *p* < 0.01; *** *p* < 0.001 vs. Q group. Q: quercetin (50 mg/kg, i.p.); PQ: 100 mg/kg i.p. P-quercetin (containing 50 mg/kg quercetin).

As a method for comparative bioavailability, the evolution of quercetin (Q) plasma concentration was studied following a single intraperitoneal bolus of natural and P-quercetin (PQ). Figure 1 shows mean quercetin plasma level curves obtained after administration of a dose of quercetin or P-quercetin. The maximum drug concentrations (𝐶_max_) observed in groups Q and PQ were 1.14 ± 1.28 μg/mL and 8.90 ± 4.62 μg/mL, respectively, i.e., a 7.8-fold increment for P-quercetin, indicating that the micellar formulation increased drug absorption.

Table 1 shows the model-independent pharmacokinetic parameters of quercetin in rats following the administration of a single bolus dose of natural quercetin or P-quercetin. The time to 𝐶_max_ was increased from 15 min (for natural quercetin) to 1 h (for P-quercetin). This delay may be attributed to the sustained release of the quercetin from the micellar formulation. Overall exposure of rats to the flavonoid was significantly higher for P-quercetin, as demonstrated by the larger area under the curve (AUC) translating into a 302.5% higher bioavailability. These results suggest that the higher solubility of the micellar formulation enhances its absorption.

### 2.2. Nephroprotective Efficacy Study

#### 2.2.1. Physiological State

**Figure 2:**
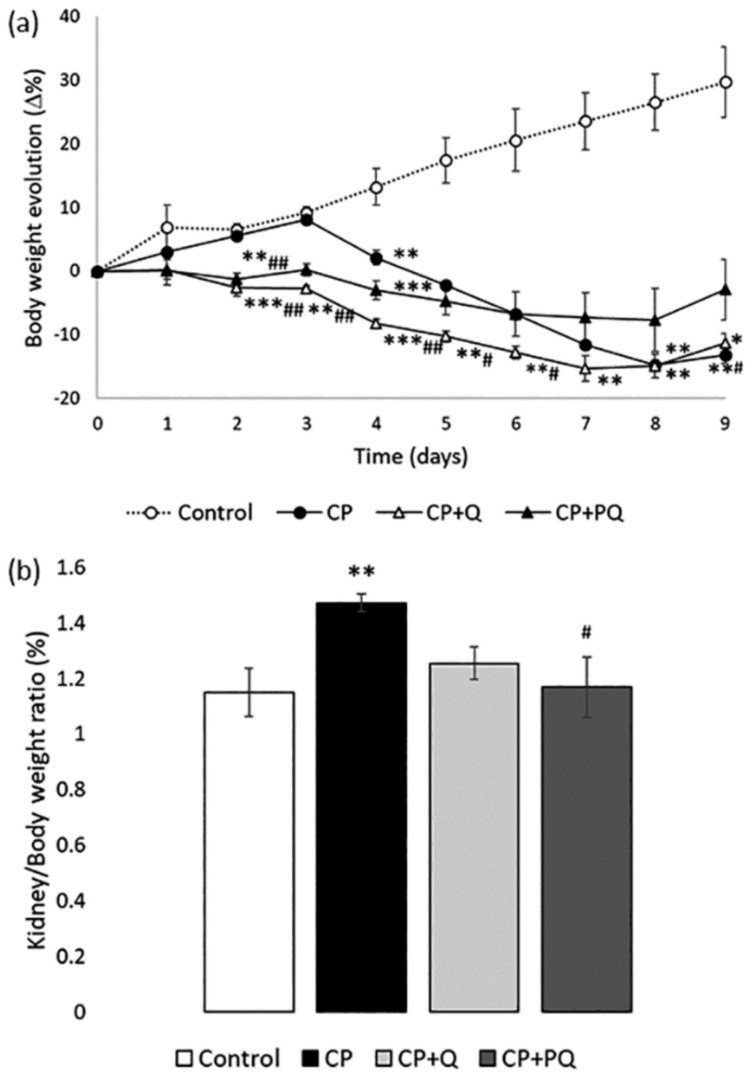
Evolution of general health status. (**a**) Percentage variation in body weight through the experiment; (**b**) Kidney/body weight ratio at day 9. Values are expressed as the mean ± SEM (*n* = 3–5 per group). * *p* < 0.05; ** *p* < 0.01; *** *p* < 0.001 vs. Control group. # *p* < 0.05; ## *p* < 0.01 vs. CP group. CP: cisplatin (6.5 mg/kg, i.p.) on day 3; CP + Q: quercetin (50 mg/kg, i.p.) for 9 days and cisplatin (6.5 mg/kg, i.p.) on day 3; CP + PQ: P-quercetin (100 mg/kg, i.p.) for 9 days and cisplatin (6.5 mg/kg, i.p.) on day 3.

As evidenced by the evolution of body weight (Figure 2a), general health deteriorated after treatment with cisplatin, compared to control animals. Neither natural quercetin nor P-quercetin significantly modified the effect of cisplatin. However, both quercetin treatments almost completely prevented the increase in the kidney/body weight ratio induced by cisplatin (Figure 2b), a parameter known to correlate with the magnitude of attrition to nephrotoxicity [32].

#### 2.2.2. Renal Function and Renal Tissue Assessment

**Figure 3:**
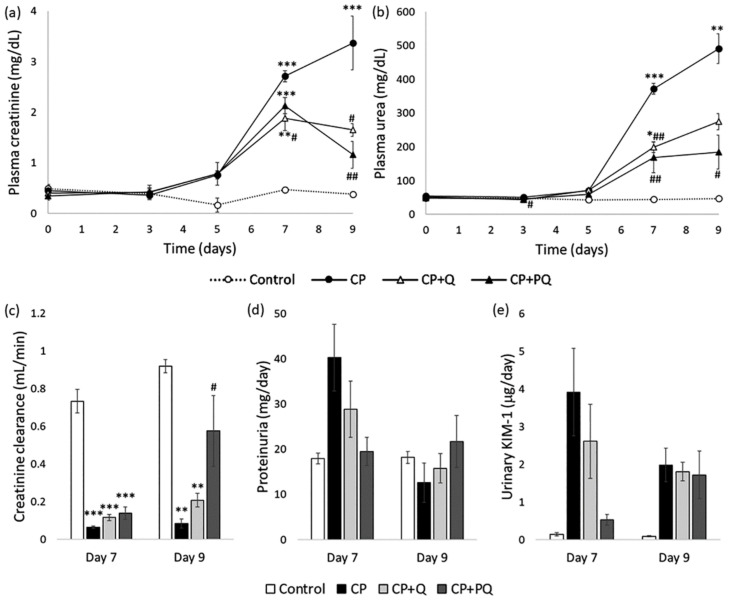
Evolution of renal parameters. (**a**) Plasma creatinine concentration; (**b**) Plasma urea concentration; (**c**) Creatinine clearance; (**d**) Proteinuria; and (**e**) KIM-1 urinary excretion. Values are expressed as the mean ± SEM (*n* = 3–5 per group). * *p* < 0.05; ** *p* < 0.01; *** *p* < 0.001 vs. Control group. # *p* < 0.05; ## *p* < 0.01 vs. CP group. CP: cisplatin (6.5 mg/kg, i.p.) on day 3; CP + Q: quercetin (50 mg/kg, i.p.) for 9 days and cisplatin (6.5 mg/kg, i.p.) on day 3; CP + PQ: P-quercetin (100 mg/kg, i.p.) for 9 days and cisplatin (6.5 mg/kg, i.p.) on day 3. KIM-1: kidney injury molecule 1.

According to international criteria, AKI is defined and diagnosed according to elevations in plasma creatinine concentration (Cr_pl_) [33,34,35], a surrogate marker of glomerular filtration rate (GFR). Other parameters, such as plasma urea concentration, are also often evaluated as azotemia indicators [36,37,38]. Increments in Cr_pl_ and plasma urea are signs of reduced GFR and AKI. In our study, renal function was severely handicapped by cisplatin, and this effect was partially ameliorated by quercetin. P-quercetin showed a slightly bolder activity than did natural quercetin, as indicated by milder damage and an improved recovery profile (Figure 3). Rats in the cisplatin (CP) group underwent an overt AKI, as they experienced a progressive and significant increase in their plasma creatinine and urea levels compared to those of the controls (Figure 3a,b). These parameters also increased in the CP + Q and CP + PQ groups, but to a significantly lower extent. Differences between the CP + Q and CP + PQ groups were not statistically significant, however, rats treated with P-quercetin showed slightly lower creatinine and urea levels than those treated with natural quercetin. Creatinine clearance (Cl_Cr_) is a standard method for GFR measurement [39,40]. In agreement with Cr_pl_ data, cisplatin induced a profound drop in, which was partially mitigated by quercetin (Figure 3c). In this case, however, a noticeable difference was seen between P-quercetin and natural quercetin, with the former being significantly more effective at improving and accelerating recovery. Of note, Cl_Cr_ and Cr_pl_ behave in an inversely proportional manner, which is only evident in the steady state. During AKI, however, renal function is continuously and rapidly changing, resulting in a slight uncoupling of this relationship.

Proteinuria is also frequently measured in the context of renal pathology. Depending on the underlying damage pattern, proteinuria may have glomerular origin (i.e., increased permeability of the glomerular filtration barrier) or, as in the case of cisplatin nephrotoxicity [13], it may arise from defective tubular reabsorption due to tubular injury. A non-significant increase in proteinuria was detected in the CP and CP-Q groups (although less marked in the latter) on day 7, which returned to normal by day 9. Similarly, the urinary excretion of a tubular damage biomarker (i.e., kidney injury molecule 1; KIM-1) [41,42] was markedly increased by cisplatin, and this increase was attenuated by both forms of quercetin, although P-quercetin was again slightly more effective.

**Figure 4:**
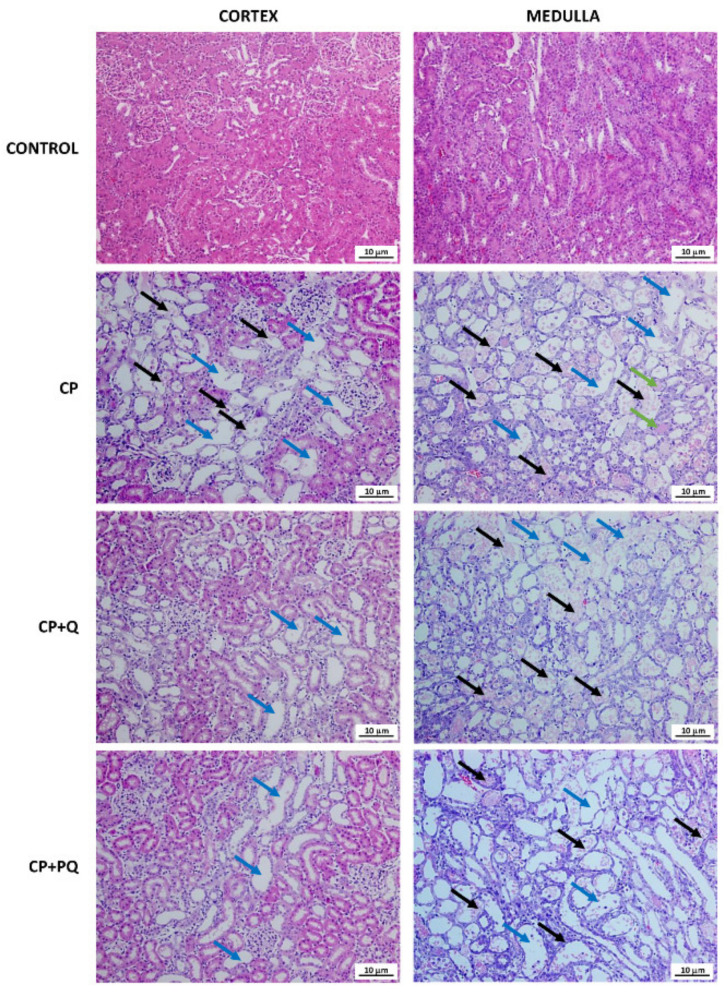
Representative images of renal specimens stained with hematoxylin and eosin. Black arrows: tubular necrosis and cell sloughing; blue arrows: tubular dilations; green arrows: intratubular deposits of hyaline material. CP: cisplatin (6.5 mg/kg, i.p.) on day 3; CP + Q: quercetin (50 mg/kg, i.p.) for 9 days and cisplatin (6.5 mg/kg, i.p.) on day 3; CP + PQ: P-quercetin (100 mg/kg, i.p.) for 9 days and cisplatin (6.5 mg/kg, i.p.) on day 3.

**Figure 5:**
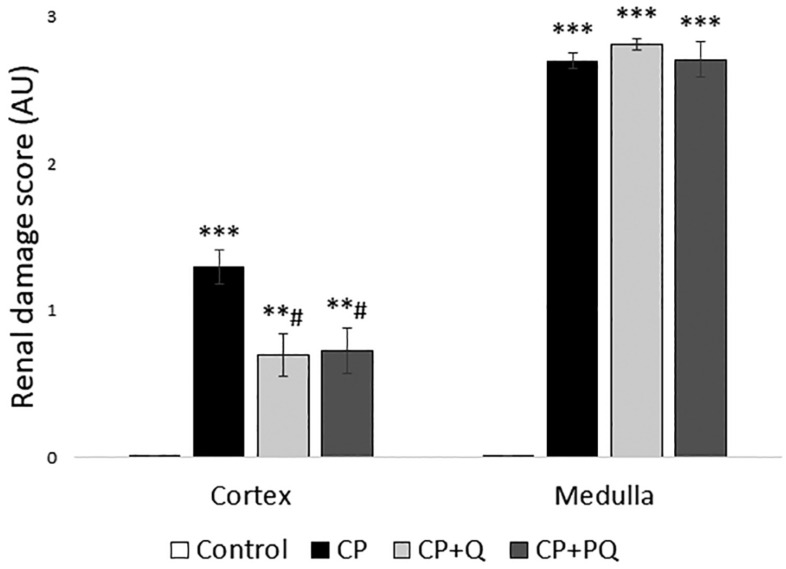
Renal damage quantification. Values are expressed as the mean ± SEM (*n* = 5 images × 3 rats per group). ** *p* < 0.01; *** *p* < 0.001 vs. Control group. # *p* < 0.05 vs. CP group. CP: cisplatin (6.5 mg/kg, i.p.) on day 3; CP + Q: quercetin (50 mg/kg, i.p.) for 9 days and cisplatin (6.5 mg/kg, i.p.) on day 3; CP + PQ: P-quercetin (100 mg/kg, i.p.) for 9 days and cisplatin (6.5 mg/kg, i.p.) on day 3. AU: arbitrary units.

The histological study of renal tissue was congruent with the biochemical findings. Specimens from rats treated with cisplatin revealed a massive tubular necrosis in the upper stripe of the outer medulla, accompanied by some cortical affection, and quercetin reduced cortical injury (Figure 4 and Figure 5). Rats that only received the nephrotoxic agent developed tubular dilation and obstruction (seen as accumulated hyaline material), and tubular necrosis with de-epithelization and cell sloughing. Both quercetin treatments similarly reduced the cortical damage induced by cisplatin, but had no effect on medullary damage (Figure 4 and Figure 5).

## 3. Discussion

Our results show that a micellar formulation of quercetin with Pluronic F127 (P-quercetin), bearing enhanced biopharmaceutical properties, increased the bioavailability of this antioxidant flavonoid and retained (or even slightly improved) its overall nephroprotective properties compared to natural quercetin.

Quercetin has been postulated as a promising candidate to protect against renal damage caused by a number of drugs and toxins, including cisplatin [25,26], methotrexate [43,44], ciprofloxacin [45], NaF [46], HgCl_2_ [47], and cadmium [48]. Although these studies demonstrated preclinical efficacy, quercetin has not been tested in similar clinical scenarios due to formulation impediments and low bioavailability, except for a clinical study in which quercetin afforded some protection from CIN [27]. Both limitations are consequences of the very poor water solubility (merely 0.01 mg/mL, at 25 °C [49]) of quercetin, and of its low stability (which is affected by temperature, pH, hydroxylation, enzymatic activity, and metal ions) [28,29,50]. Orally administered quercetin faces extensive degradation during the stomach transit due to the very low gastric pH (i.e., 1.5) [50]. In the small intestine, chemically protected by a higher pH (7.5), the remaining quercetin is only minimally absorbed. Therefore, in order to increase its bioavailability and biological efficacy, new formulations of quercetin have been developed with an aim at improving its hydrosolubility as well as protecting its active moieties from degradation, including liposomes, nanoparticles, nanoemulsions, and micelles [28].

Our micellar formulation with Pluronic F127 increases quercetin solubility ten-fold and, in in vitro studies, has shown better dissolution behavior in simulated gastric and intestinal fluids, since it achieves a significant reduction in the size of the particles and a more homogeneous dispersion of quercetin in the polymer matrix [31]. This formulation delivers a significantly increased amount of quercetin to the bloodstream, resulting in a 3-fold bioavailability that, oddly, translates only into a slightly higher nephroprotective effect. In agreement, we did not observe an additional nephroprotective effect when we used a higher dose (i.e., 100 mg/kg) of natural quercetin in previous experiments (our unpublished observations) [25,26]. In those studies, our interpretation was that, probably, higher doses of i.p. quercetin (i.e., >50 mg/kg) did not translate into increased bioavailability due to the reduced solubility, which resulted in no significantly increased net absorption. This coincided with yellow deposits of unabsorbed quercetin being found in the peritoneal cavity at sacrifice. Furthermore, our present results show that even higher plasma concentrations of quercetin (such as those obtained with P-quercetin) translate into only a slightly higher effect. Because quercetin distribution has been explained in a simplified form by a first-order, two-compartment model [51], access to target cells from the main compartment (i.e., the bloodstream) seems not to be the limitation. Thus, the reason why the maximal nephroprotective effect is almost attained with the lower plasma concentrations yielded by natural quercetin remains elusive.

This has practical implications for the further development of quercetin as a prophylactic nephroprotector. First, these results open the possibility to studying if lower dosage regimes will be similarly effective, as an ample excess of bioavailability of P-quercetin can be sacrificed without losing any therapeutic effect but will maximize the security profile. Second, the opportunity is now open for oral administration, which nonetheless needs to be tested for the new formulation. Absorption from the peritoneal cavity avoids the barriers encountered through the oral route. It is hypothesized that the higher solubility in the intestinal lumen might lead to an increased and set quercetin bioavailability within the therapeutic window. Third, the intravenous route might now be a realistic alternative with minimized toxicity, which would avoid absorption barriers. Hitherto, experimental injectable formulations of quercetin used dimethyl sulfoxide (DMSO) as a solvent [51].

Cisplatin is known to accumulate in and damage the proximal and distal tubules, and induces tubule epithelial cell apoptosis and necrosis, depending on the concentration [16]. The proximal S3 segment is most affected, although the S1 and S2 are also increasingly damaged by higher doses. In agreement with its known antiapoptotic properties on tubule cells [52,53], quercetin reduced cortical tubular de-epithelization. The identical effect seen with both formulations reinforces the idea that the quercetin distribution kinetics to the kidneys are saturated in our study. Furthermore, within the kidneys, quercetin shows a different behavior along the nephron. In fact, quercetin had no effect on the outer medulla, where the S3 segment of the proximal tubules is found. Quercetin seems to accumulate in the S1, S2, or distal tubules, which are located close to the cortex. Because it is unknown how (i.e., which transporters or diffusion pathways) and from where (i.e., the luminal or the basolateral side) quercetin accesses tubular cells, more research is necessary to explain these differential effects.

The moderate cortical tissue preservation may explain only in part the effect of quercetin on renal function (i.e., GFR). Additional protection may arise from the vascular effects of quercetin. Endothelial function participates in the regulation of renal blood flow (RBF) and GFR by modulating afferent and efferent arteriole contractile tone [54,55,56,57]. Cisplatin induces endothelial dysfunction, and this is believed to substantially contribute to the drop in GFR, along with the renal afferent vasoconstriction induced by the tubuloglomerular feedback mechanisms (activated by tubular damage) and by inflammatory cytokines [13,58]. Indeed, reversal of endothelial dysfunction is a widely recognized effect of quercetin (and of flavonoids in general) [59,60,61], which may explain why quercetin improves RBF (and thus GFR), as reported previously [25]. In addition, these endothelial-vascular effects might also help explain the slightly higher effectivity of P-quercetin at improving renal function and renal function recovery. It is probably not unreasonable to speculate that a higher bioavailability might have a bolder effect on the endothelial layer in direct contact with the blood.

Some of the effects observed after administration of quercetin might be exerted by its metabolites. Quercetin is metabolized in the intestinal mucosa and the liver by glucuronidation, sulphation, and methylation reactions [62], with the most abundant metabolites being glucuronide metabolites in the bloodstream [63]. Specifically, quercetin-3-b-O-glucuronide (Q3GA), a major plasma metabolite, has been shown to exert anti-inflammatory and vascular effects, both directly and after metabolization back to the aglycone form [64]. More research is necessary to understand the specific metabolites responsible for nephroprotection and their differential production and transformation from different administration sites to their final targets.

In conclusion, this initial study shows the therapeutic potential of P-quercetin as an improved formulation with enhanced biopharmaceutical and pharmacokinetic properties, useful for further development and prospective clinical use in the prophylaxis of nephrotoxicity.

## 4. Materials and Methods

All chemicals and reagents were purchased from Merck (Darmstadt, Germany) except where otherwise indicated.

### 4.1. Preparation of the Micellar Formulation (P-quercetin) and the Natural Quercetin Formulation

Quercetin hydrate (minimum purity of 95%) was acquired from Acros Organics (Madrid, Spain) and ethylene oxide-propylene oxide block copolymer Pluronic F127 (average molecular weight 12.6 kDa, hydrophilic-lipophilic balance 22) was provided by BASF (Ludwigshafen am Rhein, Germany). For the micellar formulation, the supercritical antisolvent precipitation technique was used to produce quercetin/Pluronic F127 particles (P-quercetin) as previously described [31]. The resulting Pluronic-quercetin formulation had a relative composition of 50%/50% w/w Pluronic F127/quercetin. For the natural quercetin formulation, quercetin was suspended in 0.16% Tween 20 in saline, as previously described [25,26].

### 4.2. Animals and Bioethics

All procedures were approved by the Bioethics Committee of the University of Salamanca and the Regional Government of Castile and Leon, Ministry of Agriculture and Livestock (code: 0000037, 27 July 2015). Animals were handled according to the guidelines of the European Community Council Directive 2010/63/UE and to the current Spanish legislation for experimental animal use and care (RD 53/2013, 01 February 2013). Male Wistar rats (200–250 g) were maintained under controlled environmental conditions within the University of Salamanca Animal House facility, with free access to water and standard chow.

### 4.3. Bioavailability Study

Rats were divided into two experimental groups: Q (*n* = 5), in which animals received a single i.p. dose of quercetin (50 mg/kg); and PQ (*n* = 5), in which animals received a single i.p. equimolar dose of P-quercetin (100 mg/kg (i.e., containing 50 mg/kg quercetin)). Subsequently, blood samples were taken in ethylenediaminetetraacetic acid (EDTA)-coated tubes from a small incision in the tail tip at the following times: 0.25, 0.5, 1, 2, 8, 12, and 24 h. The plasma was obtained by centrifugation and 10 μL of 10 mM ascorbic acid (to avoid quercetin degradation) was added to 100 μL of plasma and frozen at −80 °C until its analysis. Quercetin concentrations were determined by a reverse phase, high performance liquid chromatography (HPLC) method with UV detection. A Purospher 3 μm particle size C18 column was used, with a mobile phase composed of 28% acetonitrile and 72% of a 0.2% orthophosphoric acid water solution, at a 1 mL/min flow rate. The detection wavelength was 371 nm. Before injection in the chromatography equipment, samples were subjected to a deglucuronization process for the quantification of total quercetin. For this purpose, 1000 units of β-glucuronidase from *Helix pomatia* in 0.1 M acetate buffer (pH 5) were added to 100 μL of plasma, and this mixture was incubated at 37 °C for 1 h. Then, an extraction process was carried out with 100 μL of a 0.5 M 80:20 acetonitrile/acetic mixture (three times). Once the supernatant was evaporated in a nitrogen stream, the dry residue was re-dissolved in 40 μL mobile phase, and 20 μL were injected in the HPLC system.

### 4.4. Nephroprotection Study

**Figure 6:**
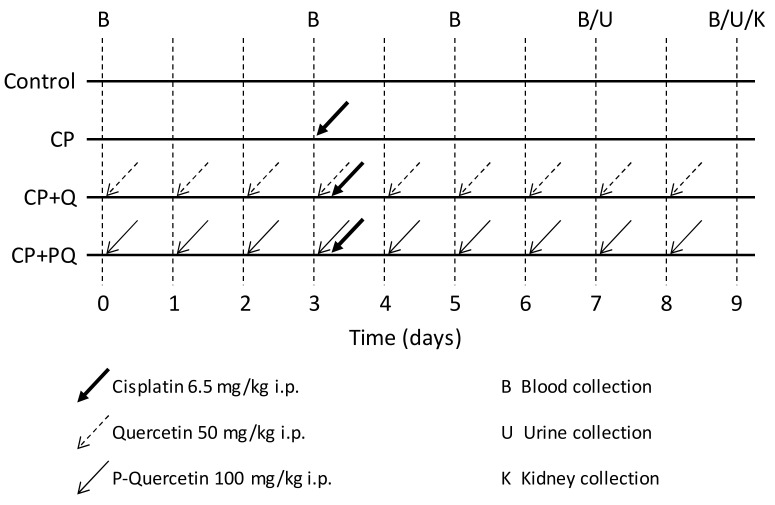
Scheme of the nephrotoxicity model. CP: cisplatin (6.5 mg/kg, i.p.) on day 3; CP + Q: quercetin (50 mg/kg, i.p.) for 9 days and cisplatin (6.5 mg/kg, i.p.) on day 3; CP + PQ: P-quercetin (100 mg/kg, i.p.) for 9 days and cisplatin (6.5 mg/kg, i.p.) on day 3.

Rats were divided into the following experimental groups (Figure 6): Control (*n* = 3) animals received vehicle (NaCl 0.9%) intraperitoneally (i.p.) for 9 days; CP (*n* = 5) animals received a single nephrotoxic dose of cisplatin (6.5 mg/kg, i.p.) on day 3 of the experiment; CP + Q (*n* = 5) animals received a daily dose of quercetin (50 mg/kg, i.p.) for 9 days and a single dose of cisplatin (6.5 mg/kg, i.p.) on day 3; and CP + PQ (*n* = 5) animals received a daily dose of P-quercetin (100 mg/kg, i.p. (i.e., containing 50 mg/kg quercetin)) for 9 days and a dose of cisplatin (6.5 mg/kg, i.p.) on day 3.

Blood samples (150 μL) were collected on days 0, 3, 5, 7, and 9 in heparinized capillaries from a small incision in the tail tip. Plasma was separated by centrifugation (11,000 rpm for 3 min) and kept at −80 °C. On days 7 and 9, 24 h urine was collected in metabolic cages, cleared by centrifugation (2000× *g* for 9 min) and stored at −80 °C. At the end of the experiment (day 9), rats were anesthetized and their kidneys were dissected, weighed, and fixed in 3.7% para-formaldehyde for histological studies.

Plasma and urine creatinine were measured using a commercial kit based on the Jaffe method [65] (QuantiChrom Creatinine Assay Kit, BioAssay Systems, Hayward, CA, USA). Plasma urea was determined using a commercial kit based on the Jung method [66] (QuantiChrom Urea Assay Kit, BioAssay Systems, Hayward, CA, USA). Creatinine clearance (Cl_Cr_) was calculated using the formula: Cl_Cr_ = Cr_ur_ × UF/ Cr_pl_; where Cr_ur_ corresponds to urinary concentration of creatinine, UF is urine flow, and Cr_pl_ is plasma concentration of creatinine. Proteinuria was measured with the Bradford assay [67]. KIM-1 was quantified using the Rat Kidney injury molecule 1 (KIM-1) ELISA Kit (Cusabio, Houston, TX, USA), following the manufacturer’s instructions.

For histological studies, renal specimens were embedded in paraffin and 5 μm tissue sections were stained with hematoxylin and eosin. Photographs were taken under an Olympus BX51 microscope connected to an Olympus DP70 color, digital camera (Olympus, Madrid, Spain). Damage quantification was performed in a blind manner as previously described [68]. In short, five random photographs of the cortical region and five photographs of the external medullary region (i.e., the areas damaged by cisplatin) were taken, evenly mapping these areas. Every image was divided into 10 identical sections (using Microsoft Office PowerPoint 2016 software), each of which was assigned a score of 0 (no damage), 1 (presence of damage in less than 1/3 of the area), 2 (presence of damage between 1/3–2/3 of the area), or 3 (presence of damage in more than 2/3 of the area). Damage was evaluated taking into account the presence of tubular necrosis and cell sloughing, tubular dilation, vacuolization, presence of hyaline deposits, and loss of the brush border.

### 4.5. Statistical Analysis

Data are presented as the mean ± standard error of the mean (SEM). Outliers were identified using the Grubbs test [69]. Normal distribution of the data was evaluated using the Shapiro–Wilk test. In the bioavailability study, the comparison between the two groups was made using the Student’s *t* test or the Mann–Whitney U test. The pharmacokinetic study was carried out by means of a model-independent analysis of the average plasma levels of quercetin. The estimated parameters to evaluate the relative bioavailability of quercetin were area under the partial curve of plasma levels (AUC)_0_^24^, area under the total curve of plasma levels (AUC)_0_^∞^, slope of the terminal phase, half-life of elimination (t_1/2_), and mean residence time (MRT). The estimation of pharmacokinetic parameters was performed by combining the trapezoidal method for the estimation of the area under the partial curve and the nonlinear regression of the terminal phase of the plasma level curve. For the nephroprotection study, an analysis of variance (ANOVA) with Scheffe’s tests or a Kruskal–Wallis test was performed for inter-group comparisons. Statistical analysis was performed with the IBM SPSS Statistics 20.0 software (International Business Machines, Armonk, NY, USA). Microsoft Office Excel and PowerPoint 2016 (Microsoft, Redmond, WA, USA) were used to create the artwork and illustrations.

## Figures and Tables

**Table 1:** Pharmacokinetic parameters after intraperitoneal administration of P-quercetin and natural quercetin (*n* = 5 per group). Q: quercetin (50 mg/kg, i.p.); PQ: P-quercetin (100 mg/kg, i.p.). AUC_0_^24^: area under the partial curve; AUC_0_^∞^: area under the total curve; MRT: mean residence time; t_1/2_: elimination half-life; λ: terminal phase slope.

Formulation	AUC_0_^24^ (µg·h/mL)	AUC_0_^∞^ (µg·h/mL)	MRT (h)	λ (h^−1^)	t_1/2_ (h)
**Q**	13.43	44.20	73.81	1.3 × 10^−2^	55.31
**PQ**	57.70	133.70	45.82	2 × 10^−2^	34.66

## Data Availability

The data presented in this study are available on request from the corresponding author.

## Abbreviations

AKI	Acute kidney injury
ANOVA	Analysis of variance
AUC	Area under the curve
AUC_0_^24^	Area under the partial curve
AUC_0_^∞^	Area under the total curve
𝐶_max_	Maximum drug concentration
Cl_Cr_	Creatinine clearance
Cr_pl_ Cr_ur_	Plasma creatinine Urinary creatinine
GFR	Glomerular filtration rate
i.p.	Intraperitoneal/intraperitoneally
KIM-1	Kidney injury molecule 1
MRT	Mean residence time
SEM	Standard error of the mean
t_1/2_	Elimination half-life
